# Global Surgery Education and Training Programmes—a Scoping Review and Taxonomy

**DOI:** 10.1007/s12262-021-03081-w

**Published:** 2021-08-25

**Authors:** Eric O’Flynn, Arbab Danial, Jakub Gajewski

**Affiliations:** 1 Institute of Global Surgery, Royal College of Surgeons in Ireland, Dublin, Ireland; 2 School of Medicine, Royal College of Surgeons in Ireland, Dublin, Ireland

**Keywords:** Global surgery, Education, Training, LMIC, Partnership

## Abstract

Global surgery is an emerging field of study and practice, aiming to respond to the worldwide unmet need for surgical care. As a relatively new concept, it is not clear that there is a common understanding of what constitutes “global surgery education and training”. This study examines the forms that global surgery education and training programmes and interventions take in practice, and proposes a classification scheme for such activities. A scoping review of published journal articles and internet websites was performed according to the PRISMA Extension for Scoping Review guidelines. PubMed MEDLINE, EMBASE and Google were searched for sources that described global surgery education and training programme. Only sources that explicitly referenced a named education programme, were surgical in nature, were international in nature, were self-described as “global surgery” and presented new information were included. Three hundred twenty-seven records were identified and 67 were ultimately included in the review. “Global surgery education and training” interventions described in the literature most commonly involved both a High-Income Country (HIC) institution and a Low- and Middle-Income Country (LMIC) institution. The literature suggests that significant current effort is directed towards academic global surgery programmes in HIC institutions and HIC surgical trainee placements in LMICs. Four categories and ten subcategories of global surgery education and training were identified. This paper provides a framework from which to study global surgery education and training. A clearer understanding of the forms that such interventions take may allow for more strategic decision making by actors in this field.

## Background

Five billion people lack access to safe, affordable, timely surgical care and 143 million extra operations per year are required in Low- and Middle- Income Countries (LMICs) to meet current surgical need [1]. Communicable diseases are declining as a percentage of the global burden of disease but conditions which could be treated by surgery are increasing, indeed an estimated 28% of the global burden of disease is surgical [2]. The term “Global surgery” has recently entered the lexicon. A commonly cited definition of the field states that global surgery “places priority on improving health outcomes and achieving health equity for all people worldwide who are affected by surgical conditions or have a need for surgical care” [3]. Another definition notes that in practice, the term is used “often with an explicit focus on LMICs” [4]. There remains, however, a lack of conceptual clarity and understanding. Abraham et al. find that the term ‘global surgery’ is not well-understood among health professionals. There is no clear consensus on what it means to be a global surgeon” [5].

Global surgery interventions take many different forms [2]. Education and training of surgical practitioners and allied healthcare workers is a key focus of national efforts to expand access to surgical care [6, 7]. It is also a key focus of international surgical partnerships. In a systematic review of global surgery partnerships between North American and LMIC institutions, Jedrzejko et al*.* [8] find that 81% of partnerships contain a surgical education or training component.

As we look at the nature of global surgery education and training, it is helpful here to distinguish between the academic, predominantly classroom-based acquisition of broad subject knowledge — referred to in this study as “education” and the learning-by-doing, predominantly hospital-based acquisition of knowledge, skills and attitudes intended to lead to specific, concrete results — referred to in this study as “training.” The overlapping concepts of education and training are discussed in detail elsewhere [9]. Although “[t]he last ten years have seen the rise of global surgery as an academic pursuit” [10], in global surgery partnerships, clinical training continues to predominate over academic education [11]. Looking specifically at North–South surgical training partnerships Greive-Price et al. found mostly “educational exchanges between HICs and LMICs” which, in general “flowed North to South… typically at the resident level, with most originating from North America and travelling to sub-Saharan Africa” [12].

It is not clear that there is a shared conception of what constitutes, and what does not constitute, education and training in global surgery. Aiming to clarify what global surgery education and training means in practice, we undertook a scoping review of available literature. A scoping review approach was chosen as such reviews “are an ideal tool to determine the scope or coverage of a body of literature on a given topic… Scoping reviews provide a useful alternative to literature reviews when clarification around a concept or theory is required” [13].

From this scoping review, we then look to categorise self-described global surgery education and training programmes and interventions. This review and taxonomy together clarify the scope of such education and training, who is being taught, and how these programmes and interventions are structured. It is hoped that this study will contribute to thoughtful global surgery education programme design.

## Methods

The study protocol was developed following the PRISMA Extension for Scoping Reviews guidelines [14].

### Eligibility Criteria

Only published journal articles and websites in the English language were included. No time limit was placed on the search; however, as the term “global surgery” is a relatively new “emerging terminology” [4], the oldest source returned in the search is from 2010 [15].

### Information Sources

PubMed MEDLINE and EMBASE databases were searched on 13/05/2021. The PubMed MEDLINE search strategy is shown in Table 1. The electronic database search was supplemented by an internet search for relevant grey literature performed on 15/06/2021. This involved searching the term “global surgery” on Google and screening all results within the first 10 search engine result pages for education and training interventions, as well as identifying webpages from personal knowledge. At each stage of screening, EO’F and AD reviewed sources separately. Where reviewers differed, JG adjudicated.

**Table 1:** PubMed MEDLINE search strategy

“global surgery”[Title/Abstract]
AND
(((“General Surgery/education”[Mesh]) OR (“Education”[Mesh])) OR (“Global Health/education”[Mesh])) OR (educat*[Title/Abstract] OR training [Title/Abstract])
AND
((“global surgery”[Title/Abstract]) AND ((((“General Surgery/education”[Mesh]) OR (“Education”[Mesh])) OR (“Global Health/education”[Mesh])) OR (educat*[Title/Abstract] OR training[Title/Abstract]))) AND ((((partnership[Title/Abstract]) OR (“International Cooperation”[Mesh])) OR (“Developing Countries”[Mesh])) OR (low middle income[Title/Abstract] OR lmic[Title/Abstract]))

This search strategy (with appropriate language) was also used for EmBASE

### Selection of Sources of Evidence

Sources were included if:

1. They explicitly and directly reference a named education or training intervention or programme.
2. The subject of the educational programme is surgical practice, education or policy.
3. The educational programme is “global” or “international” in nature — and at least one of the sites is in a low- or middle-income country.
4. The educational programme is described by the authors as a “global surgery” intervention – or any surgical (sub-)specialty variation on this, e.g. “global urology.”
5. The source presents new information, not previously or elsewhere published. Opinion, correspondence and systematic and scoping review articles were excluded.

## Results

**Fig. 1:**
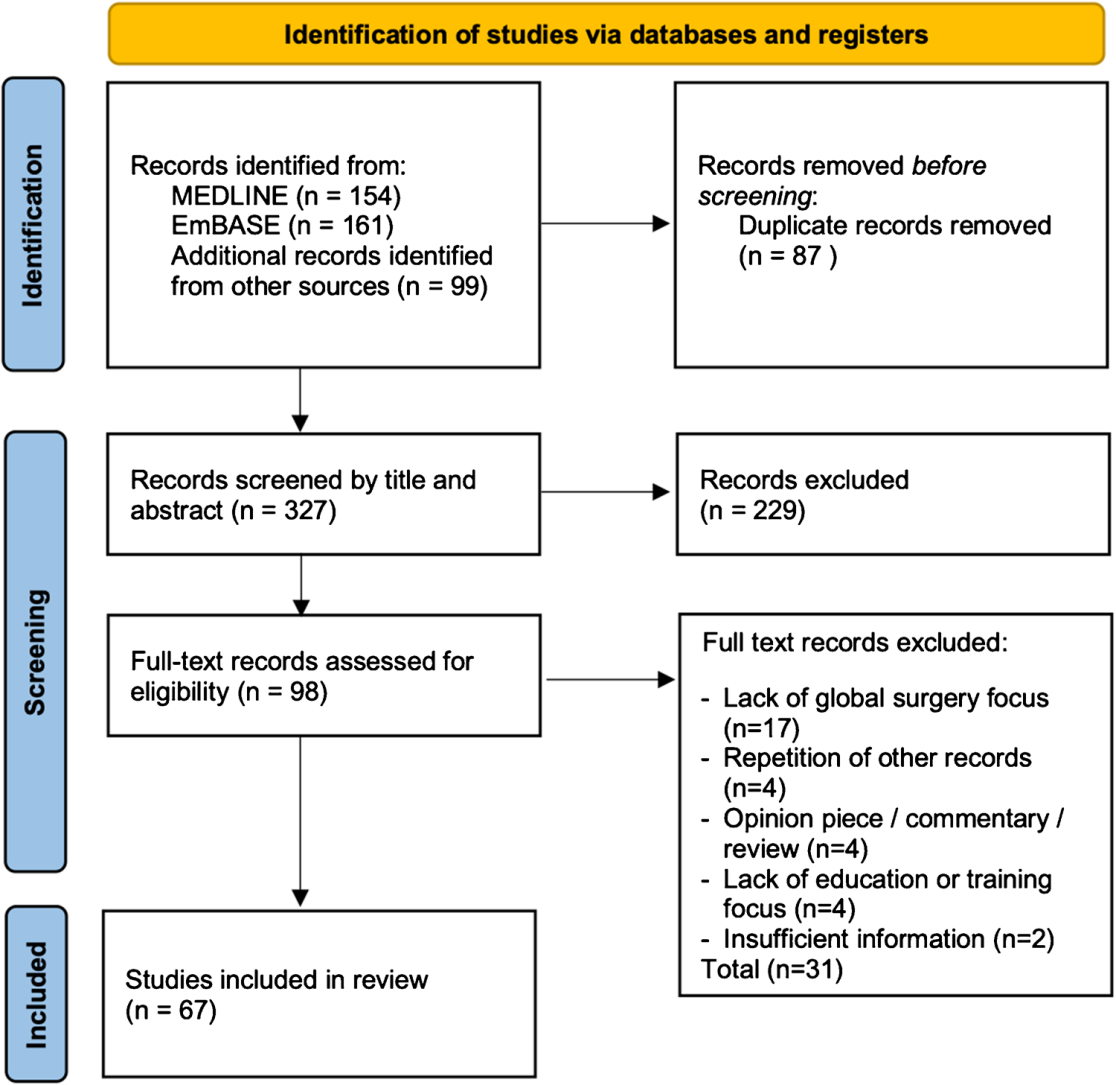
Flow chart of the literature search and screening process

Three hundred twenty-seven records were identified. Two hundred fifty-nine were excluded and 67 studies included in the review, as shown in Fig. 1. Table 2 illustrates the characteristics of the included sources. No critical appraisal of sources of evidence was undertaken.

**Table 2:** Characteristics of the included studies

No	Authors	Year	Publication type	HIC partner institution country	LMIC partner institution country	Taxonomy category	Taxonomy sub-category
1	Coleman JR, Lin Y, Shaw B, Kuwayama D	2019	Journal Article	United States	N/A	Qualified HIC surgeons	Skills before LMIC travel
2	Haji FA, Lepard JR, Davis MC, Lien ND, Can DDT, Hung CV, Thang LN, Rocque BG, Johnston JM	2021	Journal Article	United States	Vietnam	Trainee mobility	HIC to LMIC LMIC to HIC
3	Deckelbaum D.L., Gosselin-Tardif A., Ntakiyiruta G., Liberman S., Vassiliou M., Rwamasirabo E., Gasakure E., Fata P., Khwaja K., Razek T., Kyamanywa P	2014	Journal Article	Canada	Rwanda	LMIC training	Additional to formal training
4	Hayton RA, Garba LT, Teferi AN, O'Neill LR, Namm JP, Reeves ME	2019	Journal Article	United States	Malawi	Trainee mobility	HIC to LMIC
5	Cintolo-Gonzalez JA, Bedada AG, Morris J, Azzie G	2016	Journal Article	United States	Botswana	Trainee mobility	HIC to LMIC
6	Wong K, Bhama PK, d'Amour Mazimpaka J, Dusabimana R, Lee LN, Shaye DA	2018	Journal Article	United States	Rwanda	LMIC training	Additional to formal training
7	Fuller A., Tran T., Muhumuza M., Haglund M.M	2016	Journal Article	United States	Uganda	LMIC training	Supporting formal training
8	Graf J, Cook M, Schecter S, Deveney K, Hofmann P, Grey D, Akoko L, Mwanga A, Salum K, Schecter W	2018	Journal Article	Various	Tanzania	LMIC training	Supporting formal training
						Trainee mobility	HIC to LMIC
9	Behar BJ, Danso OO, Farhat B, Ativor V, Abzug J, Lalonde DH	2019	Journal Article	United States/Canada	Ghana	LMIC training	Outside formal training
10	Miller C., Haber K., Panarelli E., Samuelson R., Shahabi S	2015	Journal Article	United States	Uganda	Trainee mobility	HIC to LMIC
11	Merchant A.I., Walters C.B., Valenzuela J., McQueen K.A., May A.K	2017	Journal Article	United States	Not specified	HIC academic institution global surgery education and training	
12	Anderson G.A., Albutt K., Holmer H., Muguti G., Mbuwayesango B., Muchuweti D., Gidiri M.F., Mugapathyay S., Iverson K., Roa L., Sharma S., Jeppson B., Jönsson K., Lantz A., Saluja S., Lin Y., Citron I., Meara J.G., Hagander L	2019	Journal Article	United States/Sweden	Zimbabwe	Trainee mobility	HIC to LMIC
13	Tarpley M., Hansen E., Tarpley J.L	2013	Journal Article	United States	Kenya	Trainee mobility	HIC to LMIC
14	Love TP, Martin BM, Tubasiime R, Srinivasan J, Pollock JD, Delman KA	2015	Journal Article	United States	Haiti	Trainee mobility	HIC to LMIC
						LMIC training	Supporting formal training
15	Harvey L., Curlin H., Grimm B., Lovett B., Ulysse J.-C., Sizemore C	2020	Journal Article	United States	Haiti	LMIC training	Outside formal training
16	Jooste R, Roberts F, Mndolo S, Mabedi D, Chikumbanje S, Whitaker DK, O'Sullivan EP	2019	Journal Article	Various	Malawi	LMIC training	Outside formal training
17	Inchauste S.M., Deptula P.L., Zelones J.T., Nazerali R.S., Nguyen D.H., Lee G.K	2020	Journal Article	United States	Cuba/Vietnam	LMIC training	Outside formal training
18	Aarabi S, Smithers C, Fils MM, Godson JL, Pierre JH, Mukherjee J, Meara J, Farmer P	2015	Journal Article	United States	Haiti	Qualified HIC surgeons	LMIC Teaching placement
19	Hill K.A., Johnson E.D., Lutomia M., Puyana J.C., Lee K.K., Oduor P.R., MacLeod J.B	2018	Journal Article	United States	Kenya	LMIC training	Additional to formal training
20	Global Surgery Student Alliance—Global Surgery Curriculum	2021	Website	Various	Various	HIC academic institution global surgery education and training	
21	LeCompte M.T., Goldman C., Tarpley J.L., Tarpley M., Hansen E.N., Nthumba P.M., Terhune K.P., Kauffmann R.M	2018	Journal Article	United States	Kenya	Trainee mobility	HIC to LMIC
22	Harvard Medical School—Programme in Global Surgery and Social Change	2021	Website	United States	N/A	HIC academic institution global surgery education and training	
23	Ullrich S., Kisa P., Ruzgar N., Okello I., Oyania F., Kayima P., Kakembo N., Sekabira J., Situma M., Ozgediz D	2020	Journal Article	United States	Uganda	LMIC training	Outside formal training
24	Leeds IL, Hugar LA, Pettitt BJ, Srinivasan J, Master VA	2013	Journal Article	United States	Haiti	Trainee mobility	HIC to LMIC
25	Ellis R, Izzuddin Mohamad Nor A, Pimentil I, Bitew Z, Moore J	2017	Journal Article	United Kingdom	Ethiopia	LMIC training	Outside formal training
26	Lin Y., Scott J.W., Yi S., Taylor K.K., Ntakiyiruta G., Ntirenganya F., Banguti P., Yule S., Riviello R	2018	Journal Article	United States	Rwanda	LMIC training	Additional to formal training
27	Hendra L, Kibaja J, Kibula E, Szymankiewicz M	2020	Journal Article	United Kingdom	Tanzania	LMIC training	Outside formal training
28	Dyer GSM	2019	Journal Article	Various	Haiti	Trainee mobility	HIC to LMIC
						LMIC training	Supporting formal training
29	King’s College London—Global Health MSc	2021	Website	United Kingdom	N/A		
30	Kassam A.-F., Park C., Lungu D., Wise P.E., Mammen J.M., Benns M.V., Sussman J.J., Logan J.M	2019	Journal Article	United States	Malawi	Trainee mobility	HIC to LMIC
31	Nataraja R.M., Oo Y.M., Ljuhar D., Webb N.R., Pacilli M., Win N.N., Aye A	2020	Journal Article	Australia	Myanmar	LMIC training	HIC to LMIC
32	Davis RW, Sherif YA, Vu MT, Shilstone H, Scott B, Olutoye OO, Hollier L Jr, Nuchtern J, Rosengart TK	2021	Journal Article	United States	Ecuador/Egypt/Guatemala/Malawi /Mongolia/Tanzania/Uganda/Vietnam	HIC academic institution global surgery education and training	Outside formal training
33	Wu H.-H., Patel K.R., Caldwell A.M., Coughlin R.R., Hansen S.L., Carey J.N	2016	Journal Article	United States	Various	LMIC training	
34	Bakhshi S.K., Jooma R	2019	Journal Article	United Kingdom	Pakistan	LMIC training	Outside formal training
35	ReSurge International and COSECSA—RESURGE—COSECSA Short Term Exchange Programme	2021	Website	United States	Various – East, Central and Southern Africa	Trainee mobility	Outside formal training
36	Riviello R, Ozgediz D, Hsia RY, Azzie G, Newton M, Tarpley J	2010	Journal Article	United States	Uganda/Nigeria/Botswana/Kenya	Various	HIC to LMIC
37	Royal College of Surgeons in Ireland - Master of Surgery (by module)	2021	Website	Ireland	N/A	HIC academic institution global surgery education and training	
38	The Royal College of Surgeons in Ireland’s collaboration with COSECSA	2021	Journal Article	Ireland	Various – Sub-Saharan Africa	LMIC Training	
39	Royal College of Surgeons in England - Surgical Training for Austere Environments (STAE)	2021	Website	United Kingdom	N/A	HIC academic institution global surgery education and training	
40	Lwin AT, Lwin T, Naing P, Oo Y, Kidd D, Cerullo M, Posen J, Hlaing K, Yenokyan G, Thinn KK, Soe ZW, Stevens KA	2018	Journal Article	United States	Myanmar	LMIC training	
41	Guest GD, Scott DF, Xavier JP, Martins N, Vreede E, Chennal A, Moss D, Watters DA	2017	Journal Article	Australia	Indonesia	LMIC training	Additional to formal training
42	Mitchell KB, Giiti G, Kotecha V, Chandika A, Pryor KO, Härtl R, Gilyoma J	2013	Journal Article	United States	Tanzania	LMIC training	Supporting formal training
43	Jones CM, Campbell CA, Magee WP, Ayala R, Mackay DR	2016	Journal Article	United States	Various	LMIC training	Supporting formal training
44	McNee M.A., DeUgarte D.A., Gerstle J.T., Butler M.W., Petroze R., Holterman A.-X., Velcek F., Cleary M., Krishnaswami S., Fitzgerald T.N	2020	Journal Article	United States	Various	Trainee mobility	Supporting formal training
45	Hayton R.A., Donley D.K., Fekadu A., Woods B.K., Graybill C.K., Fitzgerald T.N	2017	Journal Article	United States	Malawi	LMIC training	LMIC to HIC
46	Butler MW, Ozgediz D, Poenaru D, Ameh E, Andrawes S, Azzie G, Borgstein E, DeUgarte DA, Elhalaby E, Ganey ME, Gerstle JT, Hansen EN, Hesse A, Lakhoo K, Krishnaswami S, Langer M, Levitt M, Meier D, Minocha A, Nwomeh BC, Abdur-Rahman LO, Rothstein D, Sekabira J	2015	Journal Article	Various	Various	Qualified HIC surgeons	Additional to formal training
47	Taro T., Yao C., Ly S., Wipfli H., Magee K., Vanderburg R., Magee W	2016	Journal Article	United States	China/Vietnam/Mexico/India	Trainee mobility	Skills before LMIC travel
						HIC academic institution global surgery education and training	
48	Sue G.R., Covington W.C., Chang J	2018	Journal Article	United States	Vietnam/Ecuador/Nepal/Zimbabwe	Trainee mobility	LMIC to HIC
49	Haider M., Jalloh M., Yin J., Diallo A., Puttkammer N., Gueye S., Niang L., Wessells H., McCammon K	2020	Journal Article	United States	Senegal	LMIC training	HIC to LMIC
50	University of British Columbia—Master of Global Surgical Care (MGSC)	2021	Website	Canada	N/A	HIC academic institution global surgery education and training	Supporting formal training
51	University of Florida - Global Surgery and Health Equity Program	2021	Website	United States	N/A	HIC academic institution global surgery education and training	
52	University of Oxford – Global Surgery	2021	Website	United Kingdom	N/A	HIC academic institution global surgery education and training	
53	Johns Hopkins University – Global Surgery Training Program	2021	Website	United States	N/A	HIC academic institution global surgery education and training	
54	University of Utah – Public Health Certificates	2021	Website	United States	N/A	HIC academic institution global surgery education and training	
55	University of Wisconsin-Madison – Global Education Opportunities	2021	Website	United States	N/A	HIC academic institution global surgery education and training	
56	Columbia University – Global Health Opportunities	2021	Website	United States	N/A	HIC academic institution global surgery education and training	
57	University of Toronto—PGME Global Health Education Initiative—Global Surgical Scholar Program	2021	Website	Canada	N/A	HIC academic institution global surgery education and training	
58	Global Surgery Amsterdam – Training Programmes	2021	Website	The Netherlands	N/A	HIC academic institution global surgery education and training	
59	University of Michigan – Global Health Research Certificate Program	2021	Website	United States	N/A	HIC academic institution global surgery education and training	
60	North-western University Feinberg School of Medicine – Institute for Global Health	2021	Website	United States	N/A	HIC academic institution global surgery education and training	
61	Baylor College of Medicine – Global Surgery Track	2021	Website	United States	N/A	HIC academic institution global surgery education and training	
62	Stanford University – Global Surgery	2021	Website	United States	N/A	HIC academic institution global surgery education and training	
63	Duke-NUS Medical School – Global Surgery Programme	2021	Website	United States	N/A	HIC academic institution global surgery education and training	
64	Yale School of Medicine – Yale Global Surgery Division	2021	Website	United States	N/A	HIC academic institution global surgery education and training	
65	Virginia Commonwealth University – VCU Program for Global Surgery	2021	Website	United States	N/A	HIC academic institution global surgery education and training	
66	McGill University—Master of Science (M.Sc.) Experimental Surgery (Thesis): Global Surgery	2021	Website	Canada	N/A	HIC academic institution global surgery education and training	
67	University of California San Francisco – Global Surgery and Public Health Pathway	2021	Website	United States	N/A	HIC academic institution global surgery education and training	

### The Meaning of Global Surgery Education and Training in Practice

This review shows that language of global surgery education and training is used to represent a narrower range of activity than definitions of global surgery aspire to [3, 4]. While *de jure* global surgery may be considered to be a “worldwide” field of study and practise in some definitions [3], de facto all programmes and interventions included in this review involve interaction between HIC and LMIC institutions, or interaction between HIC institutions and the LMIC surgical care context as the area of study and discussion.

The majority of training interventions found in our review concerned the training of surgeons. One study concerned the training of anaesthetists [16], another the surgical training of medical students [17] and two studies concerned training of different cadres of the surgical team together [18, 19]. Non-specialist cadres of surgical care provider were trained in two studies [20, 21]. Global surgery education programmes were less explicit in the cadres targeted.

Institutions from the USA were the HIC partner in 67% (*n* = 45/67) of studies. Where a single LMIC partner country was reported, East and Southern African institutions accounted for 63% (*n* = 20/32) of LMIC partners.

### Taxonomy of Global Surgery Education and Training Programmes

Our analysis of programmes which are described using the language of global surgery revealed the existence of four main categories of surgical education and training programmes and ten subcategories as shown in Table 3. Some sources reviewed describe multiple, or multi-faceted, programmes and interventions, and are thus recorded in multiple categories. The categories are as follows:

1. The academic education of surgical care providers, trainees, students, researchers and policy makers on the broad global issues related to the lack of access to safe surgical care [22–46]. A number of HIC universities and training bodies list global surgery education, training and research offerings — which take a wide variety of different forms. Among these are standalone global surgery academic courses, ranging from certificate [24, 26, 29] to master’s degree level [45], and fellowship [34]. Global surgery education may also form part of other qualifications [40, 42] or be a dedicated track in a surgical training residency [37, 39, 41, 44]. In most cases, these academic programme form part of a surgical training programme, or are targeted at surgical trainees, but in other cases open to “students and trainees” [23], “surgeons, anaesthetists and obstetricians/gynaecologists… those in training including senior medical students…[and] allied health care professions” [24], “graduate students, postgraduate trainees, surgical faculty, or surgery-related allied health care workers” [45].
2. The provision of opportunities for surgical practitioners, students and trainees to train and practice abroad. This surgical trainee mobility most commonly involved HIC surgical trainees spending time in LMIC hospitals [47–58]. Less commonly, exchanges involved LMIC surgical trainees travelling to HIC hospitals [43, 59] or bi-directional exchanges [60, 61], and in just one case a LMIC-LMIC trainee mobility programme [62].
3. HIC institutional support for surgical training in LMICS — either through support for the delivery an existing formal training programme [15, 53, 56, 58, 63–68], or additional training delivered to trainees enrolled in a formal training programme [17, 18, 48, 69–71], or delivered outside of a formal training programme [16, 19–21, 61, 72–77].
4. Provision of qualified HIC surgical care providers as trainers in LMICs [15, 61, 78], or the training of such HIC providers to work successfully in low resource environments [79, 80].

**Table 3:** Taxonomy of self-described global surgery education and training programmes and interventions

Category		Sub-category		Sources
1	HIC academic institution global surgery education and training	1		25
2	Trainee mobility	2	HIC to LMIC	12
		3	LMIC to HIC	2
		4	Bi-directional	2
		5	LMIC to LMIC	1
3	Qualified HIC surgeons	6	LMIC Teaching placement	7
		7	Skills before LMIC travel	2
4	LMIC training	8	Supporting formal training	11
		9	Additional to formal training	6
		10	Outside formal training	11

## Discussion

### Global Surgery Education and Training — a Collaborative Endeavour

All programmes and interventions included in this review involve both HIC institutions and LMIC institutions or contexts as we have described. Acknowledging that our search strategy and eligibility criteria is likely to have favoured studies that described programmes and interventions undertaken in international partnerships, nevertheless, our review of the literature suggests that surgical training and education programmes, in both HICs and LMICs, are not commonly termed as “global surgery” programmes unless they involve both HIC institutions and LMIC institutions or contexts.

### Global Surgery Education in HIC Academic Institutions

All global surgery academic education programmes identified were in HICs, aimed primarily, but not exclusively, at surgical trainees. It seems likely that these programmes are to some degree a result of HIC student and trainee demand. Patel et al. note that demand for *“*increased global surgery training reflects the enthusiasm by the current generation of HIC surgical trainees” [81]. This may be expected to benefit the well-intentioned HIC trainee; we support Patel et al.’s assertion that “Training is incomplete for any HIC surgical trainee … [who does not] have knowledge of the global context of surgical care and burden of disease.” It also seems plausible that these trainees will have a positive impact in lower resource settings than that in which they trained.

However, if we consider “knowledge of the global context of surgical care and burden of disease” [81] to be of value to LMIC students and trainees, as well as their HIC counterparts, then we must question whether such academic programmes are accessible to LMIC trainees and students, and whether there is a need or demand for such programmes in LMIC contexts. A comparison with the longer established field of global health academic education may be informative in this regard. Svadzian et al. ask “even if HIC universities made their [global health] degrees more accessible, we should still ask why an African trainee must go to London or Boston to learn [about global health].” The question may be equally pertinent when applied to academic global surgery.

### Surgical Trainee Mobility — a Two-Way Street?

Our review identified many more programmes sending HIC surgical trainees to LMICs, than bi-directional, LMIC to HIC or LMIC to LMIC programmes. This is consistent with the findings of other studies [12]. The presence of HIC surgical trainees may undoubtedly in some circumstances provide significant benefit to the LMIC host institution [48, 82]. However this apparent lack of reciprocity in travel, as similarly identified elsewhere [83], may also give us cause for concern. It is incumbent upon us as practitioners in a field dedicated to “achieving health equity for all people worldwide who are affected by surgical conditions” [3], to consider how much satisfying HIC demand for LMIC surgical placements should be a priority for the global surgical community.

### Expanding the Focus of Global Surgery Education and Training in the Literature

Global surgery may be conceived of as involving “surgeons, anaesthetists, nurses and allied healthcare professionals working together with non-clinicians, including policy makers, epidemiologists and economists” [4] but this multidisciplinarity is not currently reflected in the global surgery education and training literature. Similarly, the predominance of the USA and East and Southern Africa in the literature does not seem to reflect the global nature that the field aspires to.

### COVID 19 — an Opportunity for a Rethink

We believe the greatest surgical return on educational investment is in low-resource settings, and thus support for local training programme is likely to be highly impactful. Surgeons trained locally, stay locally [84]. Many of the models of international cooperation described in this study rely heavily on travel, which has been dramatically curtailed due to the pandemic. While hugely disruptive, this may also be “an opportunity to rethink global cooperation in higher education and research” [85]. Innovative approaches to the delivery of global surgery education and training at a distance may prove more efficient than models based on trainees and surgeons flying across the world [86]. Notable in this regard is the launch of the US$5million Global Surgical Training Challenge Prize [87], which represents a new approach to supporting LMIC surgical training innovation, leveraging expertise across the world, without any in-person meeting.

### South-South Cooperation

Trainee rotations in other low-resource environments may offer more contextually appropriate learning opportunities than rotations in high-resource environments. HIC institutions can facilitate such rotations as ReSurge is doing for plastic surgery trainees of the College of Surgeons of East, Central and Southern Africa (COSECSA) [62]. Similarly, local LMIC developed training content may be more appropriate than content developed elsewhere. HIC institutions may play a useful role here also in facilitating the creation of such resources such as the pan-African paediatric surgery e-learning platform developed by the West African College of Surgeons and COSECSA, facilitated by RCSI and KidsOR [88].

### Limitations

As the study aims to understand what it is meant by global surgery education and training through analysis of programmes using that language, it necessarily does not include studies which may be conceived of as global surgical in nature, yet which do not use the term. As an “emerging terminology” [4], the use of the term “global surgery” in relation to education and training is still evolving.

The search returned little information about programmes related to the education and training of anaesthetists, nurses, non-specialist surgical providers and allied professionals. Use of terms referring to other members of the surgical team — such as “global anaesthesia” — may produce different results.

The taxonomy produced is by necessity reductive, many global surgery education programmes may recognise their work in a number of these conceptions. Many programmes which enable HIC country trainees to spend time in Low- and Middle-Income Countries (LMICs) also provide training for such HIC trainees pre-departure, and provide reciprocal opportunities for LMIC trainees to spend time in a high-resource environment. The authors’ own institution supports formal surgical and anaesthesiology training programmes in LMICs [89], supports in-service training and capacity development outside of formal training programmes [90], and delivers academic and clinical global surgery education to students and trainees in Ireland.

## Conclusion

The growth of the global surgery paradigm presents an opportunity to leverage international cooperation to expand and enhance education and training programmes which ultimately benefit the surgical patient, worldwide. This paper provides a framework from which to further study global surgery education and training. Our review suggests that significant effort has been directed towards global surgery programmes in HIC academic institutions and HIC surgical trainee placements in LMICs. The ultimate impact of the identified categories on the surgical patient remains to be determined; however, a clearer understanding of the forms that global surgery education and training interventions take may allow for more strategic decision making by all partners.

## Data Availability

Data analysed during this study are listed in the article.
